# Assessment of Suicide Risks During the First Week Immediately After Discharge From Psychiatric Inpatient Facility

**DOI:** 10.3389/fpsyt.2021.643303

**Published:** 2021-04-20

**Authors:** Trine Madsen, Eybjørg Egilsdottir, Chanette Damgaard, Annette Erlangsen, Merete Nordentoft

**Affiliations:** ^1^Danish Research Institute for Suicide Prevention, Mental Health Centre Copenhagen, Copenhagen University Hospital – Mental Health Services, Copenhagen, Denmark; ^2^Copenhagen Research Center for Mental Health, Mental Health Centre Copenhagen, Copenhagen University Hospital – Mental Health Services, Copenhagen, Denmark; ^3^Department of Mental Health, Johns Hopkins Bloomberg School of Public Health, Baltimore, MD, United States; ^4^Research School of Public Health, Center of Mental Health Research, Australian National University, Canberra, ACT, Australia; ^5^Department of Clinical Medicine, University of Copenhagen, Copenhagen, Denmark

**Keywords:** suicide risk assessment, clinical data, psychiatric inpatients, psychiatric admission, post-discharge follow-up visit

## Abstract

**Background:** The suicide rate in first week after psychiatric discharge is alarmingly high. Although a risk assessment prior to discharge is standard praxis, it can be difficult to take into consideration the obstacles that patient will meet once discharged. A follow-up-visit during the first week after discharge is an opportunity to reevaluate whether a person may be at risk of suicide.

**Aim:** To determine how many patients, of those who were assessed, were evaluated to be at elevated risk of suicide during the first week after psychiatric discharge and secondarily to identify predictors of this and predictors for receiving a follow-up visit during first week after discharge.

**Methods:** All patients discharged between March 1st 2018 to January 17th 2019 were offered a home visit including a systematic risk assessment. Socio-demographics and clinical variables were obtained from medical records and logistic regression analyses were used to identify predictors of a higher suicide risk assessment as well as receiving a follow-up visit.

**Results:** Information from 1905 discharges were included. Of these, 1,052 were seen in follow-up meetings. Risk assessments was conducted in a total of 567 discharge procedures, of which 28 (5%) had an elevated risk of suicide. A history of suicide attempt, suicide risk having been the reason for admission, a first diagnosis of a psychiatric disorder was associated with an elevated risk of suicide after discharge.

**Conclusion:** Follow-up visits could serve as an important tool to identify people whose suicidal risk were overlooked at discharge or exposed to severe stressors after discharge.

## Background

Compared to the general population, people with mental disorders have three times higher mortality rates and 15 years shorter life spans (1–3). Excess mortality has been reported for most causes of death in people with mental disorders, but suicide is associated with the highest risk (1–3). High suicide rates have been noted among persons recently discharged from psychiatric inpatient facilities (4, 5), particularly first week after discharge is associated with excess risk (6). Although the suicide rate decline relative to time since discharge, rates remain elevated several years after an admission (5). Each suicide is a tragedy; not only for the person who lost his or her life to suicide, and for the relatives but also for the surrounding society. Recent findings suggest that as many as 3.3% of male and 6.3% of female suicides could be avoided if the excess suicide risk associated with the first week of post-discharge could be eliminated (5). It is, thus, important to address risks of suicides within this defined short-time frame through tailored interventions.

Leaving a protective and safe hospital environment to return home to a, at times, chaotic life with unpaid bills, risk of losing unemployment benefit, exposure to alcohol and drugs, conflicts in the family etc. can be a reality shock, although the personal situation and home environments, own resources, and social network may differ between patients. A large proportion of patients in Denmark return home to a socially challenging context as 18% are unemployed, 23% in early retirement, 45% have a low educational level, 16% have a primary diagnosis of alcohol- or substance misuse, 32% have a diagnosis within the schizophrenia spectrum, and 25% have an affective disorder (7).

Although important, little is known regarding risks of suicide in the time immediately after discharge (8). As a part of standard care, a suicide risk assessment is performed at the time of discharge in order to evaluate whether it is safe to send the patient home. In addition, the clinician has to take the coming hours and days into consideration, which can be difficult as suicidal thoughts and behaviors may fluctuate over time, sometimes even within a few hours (9–11). In general, people are not discharged until their situation is evaluated as being stable. It is, however, difficult to assess the impact by factors in the home environment (12). To help the patient cope with the possible challenges of returning home, an integrated effort, which supports the patient during the time immediately after discharge is needed. As discontinuity of treatment has been linked to an increased risk of suicidal behavior, this is an important aspect to address in the time after discharge (13–17).

As part of a national effort to reduce the excess mortality of people with mental disorders, the Danish parliament funded the SAFE intervention. *The purpose of SAFE is to reduce suicidal behavior during the first 6 months after discharge from psychiatric hospital and the evaluation of this effort will be addressed in a future study once follow-up data are available*. As a part of the SAFE intervention, patients were offered a home visit after discharge, which included a suicide risk assessment, thus allowing for an analysis of the proportion of outpatients, who, during the first week after discharge, were classified as being at increased risk of suicide.

### Aims

The primary aim of this study was to determine the proportion of newly discharged psychiatric patients, who were assessed to be at elevated risk of suicide during the first week after discharge. We also had secondary aims. Thus, we examined whether socio-demographic and clinical factors were associated with an elevated risk of suicide and as a follow-up contact shortly after discharge can be an important tool for assessing risk of suicide, we also examined predictors for receiving a follow-up visit in first week after discharge.

## Methods

The SAFE intervention was initiated while the patient was still in hospital and lasted until shortly after discharge. All patients were encouraged to engage their relatives in the treatment course ahead of being discharged or at the following follow-up visit. Both patient and relatives were informed regarding warning signs, which may be present prior to suicide. The SAFE intervention also included a face-to-face follow-up visit, preferably at patient's home, during first week after discharge. This was implemented to assess possible stressors after being discharged and to monitor follow-up treatment and risk of suicide as well as to review the crisis plan. SAFE was implemented and tested at the Mental Health Center Copenhagen (MHCC) in the time period March 1st 2018 to March 31st 2020. MHCC has a catchment area of 450,000 inhabitants. The center has 256 psychiatric beds in different inpatient wards and 11 specialized outpatient services, such as Flexible Assertive Community Teams (F-ACT) (18), Early Intervention Services with OPUS-teams (19, 20), team for intensive treatment of affective disorder, team for suicide prevention (21), and acute crisis teams. There are 956 fulltime employees at MHCC.

### Participants

**Figure 1:**
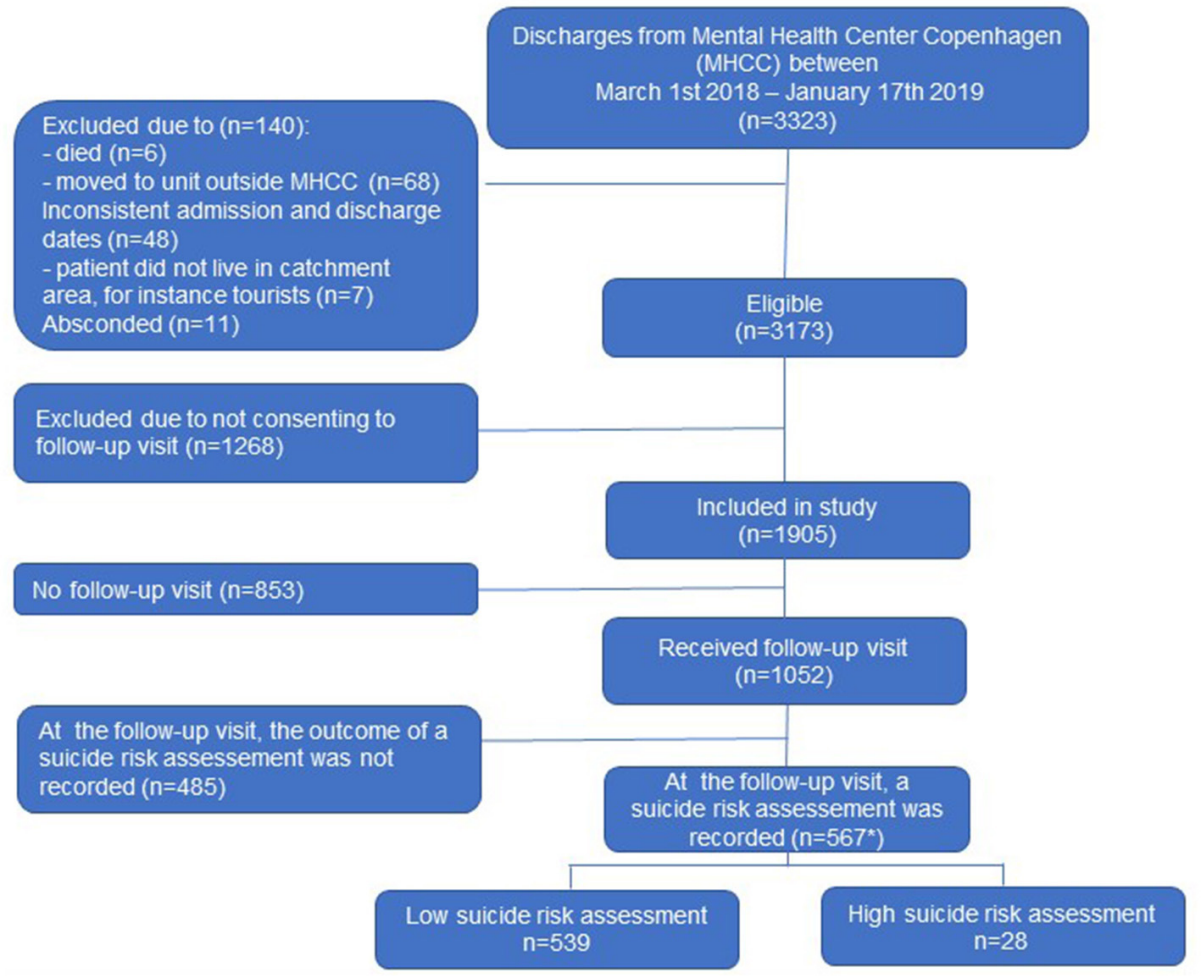
Flowchart. *In 7% of cases (*n* = 38) and 1% of cases (*n* = 8) the suicide screening assessments were, respectively carried out by telephone or information was missing on how the assessment occurred, i.e., by home visit, physical meeting elsewhere or by telephone.

Electronic medical records were obtained through the Health Platform (“Sundhedsplatformen,” a Danish version of the American EPIC system). Besides electronic medical, the outcomes of suicide risk assessments are also recorded electronically in the Health Platform, thus, allowing for linkage of these details on individual level. Information on all discharged patients aged 18 years or older from MHCC during March 1st 2018 to January 17th 2019, i.e. corresponding to largely the first 11 months of the SAFE intervention, were obtained. During this period, 3,323 discharge procedures occurred, however only in 1,905 of these discharges (corresponding to 1,205 individuals, who had between 1 and 21 admissions during the study period), did the patient consent that his/her data could be used in quality-research (see flowchart in Figure 1). There were no significant differences between patients consenting to use of data vs. those who did not with respect to sex, age, diagnoses, and days of admission.

### Outcomes

**Figure 2:**
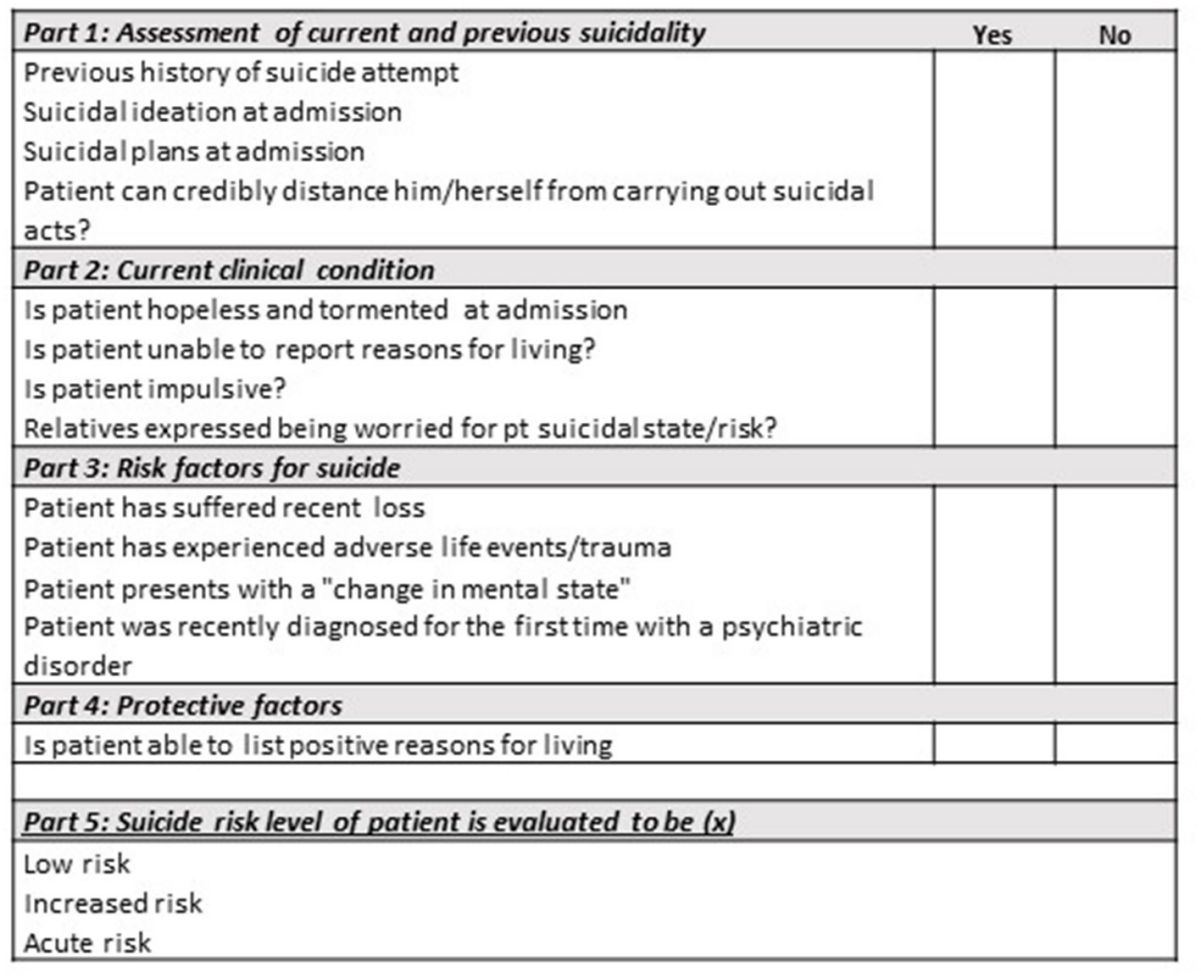
Suicide risk assessment used by MHCC (as translated into English).

The suicide risk assessment used at all clinics at the Mental Health Services in the Capital Region of Denmark guides clinicians to classify patients into 3 levels; (1) low suicide risk, (2) increased suicide risk and (3) acute suicide risk (see English translated version of the suicide risk assessment used by MHCC in Figure 2). The risk assessment is not a clinical scale based on a validated research measure, it is applied as a clinical tool intended as a checklist for the clinician. The same version of the risk assessment was used at admission, discharge and follow-up visits after discharge. The primary outcome was assessment of either an increased risk or acute suicide risk level at a follow-up visit after discharge. The secondary outcome was receiving a face-to-face follow-up visit by an outpatient psychiatric care provider during first week after discharge.

### Covariates

Data on the patients' socio-demographics and clinical characteristics were collected at admission and entered electronically into the medical records. Diagnoses, coded according to the 10th revision of the International Classification of Diseases (ICD-10), were also recorded. Following covariates were derived from the medical records: sex (male/female); age group (<30/30–49/≥50 years); schizophrenia spectrum disorder (ICD-10: F2, yes/no); affective disorder (ICD-10: F3, yes/no); anxiety- or stress-related disorder (ICD-10: F4, yes/no) and personality disorder (ICD-10: F6, yes/no); number of days admitted (1/2-3/4-14/15+ days); and day of discharge (weekday/weekend). A mandatory suicide risk assessment was conducted at time of admission. The assessment consists of 4 parts, which can be viewed as checklist-areas that the clinicians should collect patient information on; (1) the patient's current and previous suicidal behavior, (2) current clinical condition, (3) risk factors for suicide and (4) protective factors. If a patient did not have current or past suicidal behavior and his/her current clinical condition did not suggest a higher risk of suicide then part 3 and 4 could be omitted. After collecting information based on the four checklist-areas, the clinician made an overall evaluation of the patients' general condition and state of mental pain by classifying the patient risk into (1) low suicide risk, (2) increased suicide risk or (3) acute suicide risk. Finally, we had information from the medical records regarding whether patients presented at the day of admission with suicidal risk (suicidal thoughts, -plans or -threats) or due to a suicidal attempt.

### Statistical Analyses

Descriptive analyses were used to summarize patient's suicide risk assessments at follow-up. To not violate the General Data Protection Regulation (GDPR), the risk categories were combined into a binary variable, which denoted *lower risk* (low suicide risk assessment) vs. *higher risk* (increased suicide risk or acute risk level assessments). We examined in logistic regression analyses whether specific covariates were associated with a post-discharge classification of higher suicidal risk and provide odds ratios (OR) and 95% confidence intervals (95% CI). We used the SAS proc genmod procedure in the logistic regression analyses, which take into account repeated measurements from individuals who in the study period had multiple psychiatric inpatient stays.

All analyses were carried out using Statistical Analysis System (SAS studio).

## Results

During a total of 1,905 discharge procedures (1,205 individuals) between March 1st 2018 to January 17th 2019, did the patient consent that data could be used for this study. Of these, 1,052 procedures (55.2%) were seen face-to-face during follow-up meetings (415 home visits and 637 meetings at an outpatient clinic or other location). In 567 of the discharge procedures, the outcome of the suicide risk assessment was recorded. These assessments were completed during: home visits (37%), meetings in outpatient clinic/other location (55%), by telephone (7%), and missing information (1%). In those discharge procedures where the outcome of the risk assessment had been recorded, 95% (*n* = 539) were evaluated to be at lower and 5% at higher risk of suicide (*n* = 28).

### Higher Suicide Risk Assessment at Follow-Up

The association between socio-demographic and clinical factors and a higher suicide risk assessment at follow-up in the first week after discharge, was examined (Table 1). Patients with a history of suicide attempt had a 3-fold (OR = 3.11; 95% CI: 1.05–9.22) higher risk of being evaluated with a higher risk of suicide compared with those with no such history. Patients who had been admitted due to a suicide attempt were almost 5-fold (OR = 4.93; 95% CI: 1.04–23.31) as likely to be assessed as having a higher risk of suicide at follow-up after discharge vs. those not admitted with a suicide attempt. An assessment of an increased or acute suicide risk level at the time of admission was seemingly not predictive of higher suicide risk assessment at follow-up (OR = 1.55; 95% CI: 0.49–4.94). However, those who were evaluated to have a higher suicide risk at the day of discharge (*n* = 6) were also likely to have a higher risk assessment at follow-up (OR = 5.92; 95% CI: 1.43–24.51). Patients who had recently been diagnosed with a psychiatric disorder for the first time were more than 8-fold (OR = 8.58; 95% CI: 1.66-44-38) as likely to have a higher suicide risk assessment at follow-up. This was even more accentuated if the diagnosis had been an affective disorder (OR = 18.66; 95% CI: 2.04–170.9) or a personality disorder (OR = 9.25; 95% CI; 2.66–32.18). We conducted a sub-group analyses stratified by presence of any of the 6 significant predictors, i.e., history of suicide attempt, admitted due to suicide attempt, high risk at day of discharge, first diagnosis of psychiatric disorder, a diagnoses with affective disorder and a diagnosis with personality disorder. Among patients who did not present with any of these predictors, 1.9% (4/209) were evaluated to be at higher risk of suicide at the follow-up visit. Among patients who presented with one, two or three of these predictors 5.5% (13/236), 4.7% (5/106) and 37.5% (6/16), respectively, were evaluated to have a higher risk at follow-up. No one presented with four or more of the significant predictors.

**Table 1:** Univariable associations between examined variables and a higher suicidal risk assessment in 567 discharges from Mental Health Center Copenhagen.

	**Higher suicide risk assessment after discharge *n* = 28 (%)**	**Low suicide risk assessment after discharge *n* = 539 (%)**	**Univariable OR (95% CI)^#^**
**SOCIO-DEMOGRAPHICS**			
**Sex**			
Female	23 (82)	326 (60)	2.39 (0.85–6.71)
Male	5 (18)	213 (40)	1
**Age groups**			
<30 y	15 (54)	186 (35)	1.54 (0.52–4.53)
30–49 y	8 (29)	198 (37)	0.81 (0.24–2.77)
≥ 50 y	5 (18)	155 (28)	1
**SUICIDALITY**			
**Previous history of suicide attempt**			
No	<8^*^	239 (44)	1
Yes	19^*^	209 (39)	3.11 (1.05–9.22)
Missing	<3^*^	91 (17)	0.97 (0.16–5.76)
**Suicidal ideations at admission**			
No	<8^*^	200 (37)	1
Yes	20^*^	290 (54)	0.63 (0.20–2.01)
Missing	<3^*^	49 (9)	0.18 (0.01–5.50)
**Suicidal plans at admission**			
No	17^*^	320 (60)	1
Yes	<12^*^	158 (29)	0.90 (0.24–3.31)
Missing	<3^*^	61 (11)	0.22 (0.01–3.49)
**Admitted with suicidal risk**			
No	4 (14)	199 (37)	1
Yes	12 (43)	187 (35)	1.59 (0.36–7.04)
Missing	12 (43)	153 (28)	3.51 (0.96–12.84)
**Admitted due to a suicide attempt**			
No	12 (43)	358 (66)	1
Yes	4 (14)	28 (5)	4.93 (1.04–23.31)
Missing	12 (43)	153 (28)	3.33 (1.16–9.59)
**Patient can credible distance him/her self from carrying out suicidal acts?**			
No	<8^*^	110 (20)	1.32 (0.42–4.15)
Yes	19^*^	367 (68))	1
Missing	<3^*^	62 (12)	0.51 (0.07–3.55)
**Suicide risk assessment at day of admission**			
Low	14^*^	359 (67)	1
Increased/acute	<14^*^	145 (27)	1.55 (0.49–4.94)
Missing	<3^*^	35 (6)	0.34 (0.01–20.67)
**Suicide risk assessment at day of discharge**			
Low	22^*^	424 (79)	1
Increased/acute	<3^*^	4 (1)	5.92 (1.43–24.51)
Missing	<4^*^	111 (20)	1.00 (0.08–12.08)
**PREDICTORS FROM THE SUICIDE RISK ASSESSMENT**			
**Is patient hopeless and tormented at admission?**			
No	14^*^	210 (39)	1
Yes	<14^*^	246 (46)	0.83 (0.28–2.44)
Missing	<3^*^	83 (15)	0.17 (0.01–2.19)
**Is patient unable to report reasons for living?**			
No	23^*^	363 (67)	1
Yes	<4^*^	81 (15)	0.32 (0.03–3.38)
Missing	<3^*^	95 (18)	0.31 (0.06–1.72)
**Is patient impulsive?**			
No	13 (46)	273 (51)	1
Yes	9 (32)	151 (28)	1.21 (0.31–4.66)
Missing	6 (22)	115 (21)	0.63 (0.13–3.19)
**Relatives worried for patient's suicidal state/risk?**			
No	14 (50)	238 (44)	1
Yes	4 (14)	128 (24)	1.14 (0.05–26.26)
Missing	10 (36)	173 (32)	0.90 (0.21–3.93)
**Patient suffered recent loss?**			
No	22^*^	397 (74)	1
Yes	<3^*^	27 (5)	0.78 (0.04–14.28)
Missing	<5^*^	115 (21)	0.35 (0.05–2.32)
**Patient experienced adverse life events/trauma?**			
No	21 (75)	324 (60)	1
Yes	4 (14)	81 (15)	0.60 (0.17–2.06)
Missing	3 (11)	134 (25)	0.18 (0.02–2.00)
**Patient appears with “change in mental state”?**			
No	18 (64)	234 (43)	1
Yes	7 (25)	176 (33)	0.51 (0.13–1.96)
Missing	3 (11)	129 (24)	0.25 (0.05–1.39)
**Is patient recently diagnosed for first time with psychiatric disorder?**			
No	19^*^	382 (71)	1
Yes	<3^*^	19 (4)	8.58 (1.66–44.38)
Missing	<8^*^	138 (26)	0.82 (0.24–2.79)
**Patient report positive reasons for living?**			
No	4 (14)	76 (14)	1
Yes	18 (64)	323 (60)	2.29 (0.13–39.07)
Missing	6 (22)	140 (26)	1.43 (0.08–25.32)
**CLINICAL VARIABLES**			
**Schizoprhenia spectrum disorder**			
No	21 (75)	339 (63)	1
Yes	7 (25)	200 (37)	0.01 (0.00–509.84)
**Affective disorders**			
No	21 (75)	372 (69)	1
Yes	7 (21)	167 (31)	18.66 (2.04–170.94)
**Anxiety– or stress–realted disorder**			
No	23 (82)	468 (87)	1
Yes	5 (18)	71 (13)	0.94 (0.15–5.89)
**Personality disorder**			
No	21 (75)	511 (95)	1
Yes	7 (25)	28 (5)	9.25 (2.66–32.18)
**Duration of hospital admission**			
1 day	10 (36)	113 (21)	2.36 (0.60–9.32)
2 to 3 days	7 (25)	78 (14)	1.40 (0.11–17.54)
4 to 14 days	4 (14)	110 (20)	1.36 (0.26–7.24)
≥15 days	7 (25)	238 (44)	1
**Day of discharge**			
Weekend	7 (25)	44 (8)	1
Weekday	21 (75)	495 (92)	0.34 (0.05–2.21)

* *To not violate the General Data Protection Regulation (GDPR) column percentages are not presented*.

# *Odds Ratios were adjusted for multiple risk assessments of the same individual*.

### Receiving a Face-to-Face Follow-Up Visit After Discharge

Table 2 presents which factors predicted receiving a follow-up visit during the first week of discharge in those 1,905 discharge procedures that were examined in the study. Females were more likely than males to receive a follow-up visit (OR = 1.35; 95% CI: 1.11–1.66). Patients who felt hopeless or appeared tormented at the admission had a higher probability (OR = 1.39; 95% CI; 1.13–1.72) of receiving a follow-up visit. The same applied for those whose relatives had uttered concerns that the patient might be suicidal (OR = 1.38; 95% CI: 1.05–1.82). Also, patients who presented with suicidal thoughts or plans upon admission were more likely to receive a follow-up visit (OR = 1.39; 95% CI: 1.08–1.80). A follow-up visit was also more likely to take place if the patient had been diagnosed with a schizophrenia spectrum disorder (OR = 1.43; 95% CI: 1.14–1.80) or an affective disorder (OR = 1.71; 95% CI: 1.33–2.19). Patients who had been hospitalized for more than 1 day (reference), were also more likely to receive a follow-up visit. In fact, a trend suggested that longer hospital stays increased chances of receiving a follow-up visit. Finally, a follow-up visit was 58% (OR = 1.58; 95% CI: 1.21–2.07) more likely to take place if the patient had been discharged on a weekday vs. in the weekend.

**Table 2:** Univariable associations between examined variables and receiving a physical visit in first week after discharge in 1905 discharges from Mental Health Center Copenhagen.

	**Visit *n* = 1,052 (%)**	**No visit *n* = 853 (%)**	**Total *n* = 1,905**	**Univariable OR (95% CI)^#^**
**SOCIO–DEMOGRAPHICS**				
**Sex**				
Female	568 (54)	394 (46)	962	1.35 (1.11–1.66)
Male	484 (46)	459 (54)	943	1
**Age groups**				
<30 y	302 (29)	224 (26)	526	1.16 (0.89–1.51)
30–49 y	432 (41)	328 (38)	760	1.15 (0.90–1.45)
≥ 50 y	318 (30)	301 (35)	619	1
**SUICIDALITY**				
**Previous history of suicide attempt**				
No	452 (43)	380 (45)	832	1
Yes	350 (33)	267 (31)	617	1.24 (0.98–1.55)
Missing	250 (24)	206 (24)	456	1.20 (0.94–1.55)
**Suicidal ideations at admission**				
No	458 (44)	391 (46)	849	1
Yes	455 (43)	330 (39)	785	1.17 (0.95–1.43)
Missing	139 (13)	132 (15)	271	1.07 (0.79–1.43)
**Suicidal plans at admission**				
No	660 (63)	526 (62)	1186	1
Yes	242 (23)	173 (20)	242	1.12 (0.89–1.42)
Missing	150 (14)	154 (18)	150	0.94 (0.72–1.24)
**Admitted with suicidal risk**				
No	513 (49)	383 (45)	896	1
Yes	310 (45)	146 (17)	456	1.39 (1.08–1.80)
Missing	229 (22)	324 (38)	553	0.38 (0.30–0.47)
**Admitted due to a suicide attempt**				
No	777 (74)	496 (58)	1273	1
Yes	46 (4)	33 (4)	79	0.65 (0.41–1.02)
Missing	229 (22)	324 (38)	553	0.33 (0.27–0.40)
**Patient can credible distance him/her self from carrying out suicidal acts?**				
No	178 (17)	130 (15)	308	1
Yes	706 (67)	562 (66)	1268	1.11 (0.86–1.43)
Missing	168 (16)	161 (19)	329	0.97 (0.75–1.26)
**Suicide risk assessment at day of admission**				
Low	727 (69)	573 (67)	1300	1
Increased/acute	230 (22)	178 (21)	408	1.03 (0.82–1.30)
Missing	95 (9)	102 (12)	197	0.92 (0.67–1.26)
**Suicide risk assessment at day of discharge**				
Low	780 (74)	589 (69)	1369	1
Increased/acute	13 (1)	8 (1)	21	2.00 (0.89–4.49)
Missing	259 (25)	256 (30)	515	0.82 (0.66–1.02)
**PREDICTORS FROM THE SUICIDE RISK ASSESSMENT**				
**Is patient hopeless and tormented at admission?**				
No	413 (39)	385 (45)	798	1
Yes	414 (39)	273 (32)	414	1.39 (1.13–1.72)
Missing	225 (21)	195 (23)	225	1.27 (0.98–1.64)
**Is patient unable to report reasons for living?**				
No	673 (64)	548 (64)	1221	1
Yes	118 (11)	94 (11)	212	1.03 (0.76–1.38)
Missing	261 (25)	211 (25)	261	1.20 (0.95–1.52)
**Is patient impulsive?**				
No	502 (48)	389 (46)	891	1
Yes	255 (24)	228 (27)	483	0.88 (0.70–1.09)
Missing	295 (28)	236 (28)	295	1.11 (0.88–1.39)
**Relatives worried for patient's suicidal state/risk?**				
No	458 (44)	408 (48)	866	1
Yes	185 (18)	113 (13)	298	1.38 (1.05–1.82)
Missing	409 (39)	332 (39)	741	1.21 (0.99–1.49)
**Patient suffered recent loss?**				
No	619 (59)	558 (65)	1268	1
Yes	42 (4)	41 (5)	83	0.75 (0.49–1.16)
Missing	300 (29)	254 (30)	554	1.09 (0.87–1.36)
**Patient experienced adverse life events/trauma?**				
No	619 (59)	482 (57)	1101	1
Yes	113 (11)	92 (11)	205	0.85 (0.62–1.15)
Missing	320 (30)	279 (33)	599	1.00 (0.81–1.24)
**Patient appears with “change in mental state”?**				
No	446 (42)	382 (45)	828	1
Yes	296 (28)	196 (23)	492	1.02 (0.82–1.27)
Missing	310 (29)	275 (32)	585	0.82 (0.67–1.00)
**Patient recently diagnosed for first time with psychiatric disorder?**				
No	694 (66)	541 (63)	1235	1
Yes	28 (3)	25 (3)	53	0.79 (0.46–1.34)
Missing	330 (31)	287 (34)	617	1.01 (0.82–1.25)
**Patient report positive reasons for living?**				
No	116 (11)	95 (11)	211	1
Yes	578 (55)	470 (55)	1048	0.95 (0.71–1.28)
Missing	358 (34)	288 (34)	646	1.15 (0.84–1.57)
**CLINICAL VARIABLES**				
**Schizoprhenia spectrum disorder**				
No	627 (60)	557 (65)	1184	1
Yes	425 (40)	296 (35)	721	1.43 (1.14–1.80)
**Affective disorders**				
No	792 (75)	721 (85)	1513	1
Yes	260 (25)	132 (15)	392	1.71 (1.33–2.19)
**Anxiety- or stress-realted disorder**				
No	955 (91)	774 (91)	1729	1
Yes	97 (9)	79 (9)	176	0.84 (0.60–1.17)
**Personality disorder**				
No	996 (95)	810 (95)	1806	1
Yes	56 (5)	43 (5)	99	1.00 (1.00–1.00)
**Duration of hospital admission**				
1 day	232 (22)	298 (35)	530	1
2 to 4 days	213 (20)	191 (22)	203	1.55 (1.18–2.04)
5–7 days	58 (6)	67 (8)	125	1.59 (1.04–2.42)
8–14 days	91 (9)	67 (8)	158	1.72 (1.21–2.46)
*≥ 15 days*	458 (44)	230 (27)	688	2.37 (1.84–3.06)
**Day of discharge**				
Weekend	99 (9)	127 (15)	226	1
Weekday	953 (91)	726 (85)	1679	1.58 (1.21–2.07)

# *Odds Ratios were adjusted for multiple risk assessments of the same individual*.

## Discussion

To the best of our knowledge this is the first study to report findings from clinical assessments of suicide risks during the first week after discharge from psychiatric hospital. Among those patients whose suicide risk was assessed during a follow-up visit, 5 % were considered to be at high risk. Factors, such as previous suicide attempt, suicide attempt upon admission, first psychiatric diagnosis, depression, and personality disorder, were associated with a higher suicide risk assessment at follow-up. Moreover, patients whose admission was surrounded by concerns or torment were more likely to receive a follow-up visit as were those who were discharged during weekdays vs. weekends.

Although low in absolute terms, i.e., 0.12% and 0.06% of men and women die by suicide during the first week after being discharged from psychiatric hospital, in relative terms, recent discharge is a high risk period for suicide (5). Furthermore, rates of deliberate self-harm is estimated to be at least eight times higher than for suicide death (22). This study found that as many as 5% were considered as being at a higher risk of suicide during first week after discharge. Clinically, if these 5% overlap with those who actually carry out suicidal acts during this period then the SAFE intervention constitutes an important contribution to preventing suicidal behavior. The follow-up visit might, furthermore, be an opportunity to identify persons who are at risk of suicide that might have been overlooked or not emerged at the time of discharge. The follow-up visit should preferably take place in the patient's home as this will provide the clinician with additional information on the patient's mental well-being and social situation after discharge. It will also be an opportunity for assessing risks, in order to determine whether more support might be needed in the initial phase or whether a re-admission should be considered. Based on the finding that 5% were assessed with higher risk of suicide in the first week after discharge, we would recommend a follow-up visit to capture suicidal risk overseen at discharge or developed thereafter.

Patients with an affective- or personality disorder were more likely to be evaluated as being at higher risk of suicide post discharge. In contrast, patients with personality disorders were less likely to receive a follow-up visit. This discrepancy might be explained by different clinical care procedures for different groups; for years, specialized F-ACT and OPUS teams have been serving patients with severe mental disorders and often involving follow-up visits shortly after discharge (18–20). The same procedure has not been standard practice for other patient groups, such as those with personality disorders. In OPUS, which was implemented since 2002, all patients with a first episode of psychosis are teamed up with a personal contact person who accompanies them through outpatient and inpatient treatments with weekly face-to-face meetings. It is, furthermore, possible that this effort has contributed to a lower suicide risk for this patient group (5, 23).

A very recent published study from England showed that patients who died by suicide within the first 3 days after psychiatric discharge were characterized by having personality disorders (24). In alignment with this we found that patients with affective or personality disorders were associated with a higher suicide risk assessment in the first week after discharge. In a Danish setting patients discharged with these disorders were usually (before the SAFE intervention) discharged with a referral to individual- or group-based psychotherapy in outpatient settings. This type of care is, unfortunately, often only available after several weeks due to wait lists. A systematic follow-up visit for this vulnerable patient group would therefore be essential but may take time to implement. The fact that patients who were discharged after a short admission or during weekends were less likely to receive a follow-up visit might be due to neglect of the discharge procedures.

Another important finding was that patients, although only few, who at the day of discharge had been evaluated to be at a high risk of suicide also were almost 6-fold as likely to be assessed with a higher risk at the time of the follow-up visit. This may not seem surprising, and it is possible that they constitute a small proportion of patients who are chronically suicidal (25–27). At times, it is judged necessary to send home patients. However, our findings provide an important argument for ensuring that these patients are monitored closely through follow-up visits, especially if they presented with more than two of the significant predictors of a higher risk assessment after discharge as a high proportion of these (37.5%) were found to be assessed with a higher suicide risk level at the follow-up visit. In line with this, we found that those who had been admitted due to a suicide attempt were most likely to receive a follow-up visit. The same was found for patients, who reported to feel hopeless and tormented, or who had relatives expressing worries regarding the patient's suicidal state. Thus, supporting the notion that patients where a suicidal concern had been noted during the admission were more likely to receive a follow-up visit. Finally, we found that females had higher odds of receiving a follow-up visit after discharge, which follows the general pattern of a higher compliance and help-seeking behavior among females (28, 29).

The goal of the SAFE-intervention was to ensure that all patients would receive a follow-up visit, preferably at home, after discharge. Due to implementation difficulties, some patients might not have been offered a follow-up visit, while others might have declined. In a recent qualitative study aiming at understanding psychiatric inpatients' beliefs about suicide risk and post discharge follow-up treatment, 15 out of 16 patients acknowledged that they preferred seeing the provider face-to-face, because “the provider sees your emotions and can tell your affect and you won't get that on the phone” (30), supporting the notion that patients also might prefer personal follow-up after discharge. In this same study, some patients reported that if they experienced a worsening in their mental health symptoms after discharge, they would be likely to disengage from follow-up care. It is, therefore, essential to plan a follow-up visit (as in the SAFE intervention) already prior to discharge, as this gives the clinicians in the outpatient care an important, and already agreed upon, opportunity to visit the patient.

### Strengths and Limitations

This study had several strengths and limitations. Strengths include a large group of patients and a pragmatic clinical study cohort, which resembles real clinical practice. The clinical suicide risk assessment has been used for several years in clinical practice in Mental Health Services in the Capital Region of Denmark. It was originally constructed by an expert group, who compiled questions covered in different suicide risk assessment scales into a short instrument. It is not a scale, but a checklist, and the final evaluation of suicide risk is based on all available information and the clinician's overall evaluation of the patient's condition. However, a number of limitations should be mentioned. First, a large proportion of patient did not consent to use of their data for research. Although they did not differ with regard to sex, age, diagnoses and days of admission from those who provided consent, it did limit the sample size. Clinicians had to actively ask for consent for participation, which sometimes was forgotten in a busy clinical setting, and might have contributed to a lower participation rate. Missing data from the risk assessment conducted at admission and the relatively low number of risk assessments conducted during follow-up, impacted our ability to generate precise estimates, which was shown by the wide confidence intervals. Secondly, the suicide risk assessment is not based on a validated research measure, hence, assessment of inter-rater reliability is not feasible, i.e., it has not been validated whether all clinicians correctly identified existing risk factors. Likewise, some of the risk factors on the checklist were permanent, once occurred; for instance, prior suicide attempt and suicide attempt leading to admission. We cannot exclude that this might have led to patients with one of these risk factors repeatedly being evaluated as having high risk of suicide. Although our data is subject to a high number of drop out, we believe the findings from Table 1 suggest that clinicians mainly based their evaluation on the patients' current clinical and general condition. Out of those 228 registered with a prior suicide attempt at admission, 19 (8%) was evaluated as in high risk at follow-up, similarly this number was 4 (13%) out of the 32 admitted due to a suicide attempt as well as it is indicated by the difference in the proportion being evaluated as in high risk at admission (30%) vs. at follow-up (5%). A third limitation concerned the analyses of predictors for receiving a follow-up visit; we only had data for the start-up phase of the SAFE intervention, i.e., in March 2018, at the beginning of the study only 28% received a follow-up visit whereas 74% received one in December 2018. For example, we found that patients discharged with personality disorders had a lower chance of receiving a follow-up visit. This might be explained by the fact that outpatient clinics addressing patients with personality disorders had difficulties implementing the SAFE-intervention during the start-up phase vs. those outpatient clinics that had already offered follow-up visits as standard care for years. By now, hopefully all patient groups, irrespective of diagnosis, are offered a follow-up visit. Another limitation related to the latter point is that we lacked data on how many patients were offered a SAFE follow-up visit but declined this. Finally, information on newly emerged suicide risk factors occurring in the patients' life after discharge was not available thus could not be taken into account in the analyses of who is at risk of a higher suicide risk assessment after discharge, which may also be viewed as a limitation.

## Conclusions

A total of 5% of those with a registered suicide risk assessment were assessed to have a higher suicide risk at a follow-up visit during first week after discharge. Factors associated with being at risk included history of suicide attempt, suicide attempt upon admission, first psychiatric diagnosis, depression, and personality disorder. A follow-up visit was more likely to take place among patients who had been discharged during weekdays than weekends. A subsequent follow-up visit, ideally as a home visit, after discharge may help identify individuals at risk of suicide who might have been overlooked or not emerged at discharge and, thus, possibly mitigate obstacles, which arise after being discharged.

## Data Availability Statement

The datasets presented in this article are not available because the project and data was restricted regarding the rules of using quality assurance data.

## Ethics Statement

Ethical review and approval was not required for the study on human participants in accordance with the local legislation and institutional requirements. Written informed consent for participation was given for usage of quality assurance data, thus this study is in accordance with the national legislation and the institutional requirements.

## Author Contributions

Material preparation and analysis were performed by TM, EE, and CD. The draft of the manuscript was primarily written by TM and MN and all authors commented on the manuscript. All authors contributed to the article and approved the submitted version.

## Conflict of Interest

The authors declare that the research was conducted in the absence of any commercial or financial relationships that could be construed as a potential conflict of interest.
